# Synthesis of a Free-Standing Ternary WO_3_/CNT/ZnO–Chitosan Composite Photocatalytic Membrane for the Mitigation of Protein Fouling in Membranes

**DOI:** 10.3390/polym17040437

**Published:** 2025-02-07

**Authors:** Wei Tze Chong, Sze Mun Lam, Yit Thai Ong, Trong-Ming Don

**Affiliations:** 1 Faculty of Engineering and Green Technology, Universiti Tunku Abdul Rahman, Jalan Universiti, Bandar Barat, Kampar 31900, Perak Darul Ridzuan, Malaysia; chongweitze@gmail.com (W.T.C.); lamsm@utar.edu.my (S.M.L.); 2 Centre for Environment and Green Technology, Universiti Tunku Abdul Rahman, Jalan Universiti, Bandar Barat, Kampar 31900, Perak Darul Ridzuan, Malaysia; 3 Department of Chemical and Materials Engineering, Tamkang University, New Taipei City 25137, Taiwan

**Keywords:** membrane filtration, membrane fouling, free standing, ternary WO_3_/CNT/ZnO–chitosan composite, photocatalytic membrane, bovine serum albumin, antifouling

## Abstract

The application of membrane filtration, particularly micro- and ultra-filtration, in food and pharmaceutical industries often faces the issue of protein fouling. In this study, we aimed to fabricate a free-standing ternary tungsten trioxide/carbon nanotube/zinc oxide (WO_3_/CNT/ZnO)–chitosan composite photocatalytic membrane via wet processing and infiltration techniques to address the fouling issue. Infiltration with low molecular weight chitosan was found to enhance the mechanical stability of the ternary composite photocatalytic membrane. The ternary composite photocatalytic membrane with a 0.16 g ternary photocatalyst load demonstrated 86% efficiency in the degradation of bovine serum albumin (BSA) under sunlight irradiation for 120 min. A reduction in permeation flux accompanied by an increase in BSA rejection was observed as the loading of the ternary photocatalyst in the ternary composite photocatalytic membrane was increased. This can be associated with the decreased average porosity and mean pore radius. The ternary composite photocatalytic membrane demonstrated reasonably good antifouling behavior with an *R_fr_* of 82% and an *R_if_* of 18%. The antifouling property demonstrated by the ternary composite photocatalytic membrane is important in maintaining the reusability of the membrane.

## 1. Introduction

Membrane filtration processes, particularly micro- and ultra-filtration, have been extensively applied in water and wastewater treatment across various industries, including food processing and pharmaceuticals, due to their operational simplicity, energy efficiency, and high separation performance [1,2]. Additionally, these processes are widely used in the dairy industry for concentration and purification purposes, such as pre-concentrating milk, milk dehydration, whey protein fractionation and purification, and casein enrichment during milk production [3,4,5,6]. Protein is one of the main components that are present in the feed stream of these separation processes. Both microfiltration and ultrafiltration have demonstrated promising capabilities in rejecting and retaining protein molecules through their microporous structures. However, protein molecules tend to form physical and electrochemical interactions with the membrane or other components in the feed stream. This often causes the adsorption and deposition of proteins onto the membrane surface, resulting in membrane fouling. The membrane fouling can be characterized by a progressive decline in permeation flux over time under constant pressure or by an increase in transmembrane pressure. This phenomenon eventually leads to adverse effects, including reduced separation performance, increased operating costs and shortening of the membrane’s lifespan.

Extensive efforts have been made to mitigate membrane fouling, including the integration of external forces such as electric fields [7] and sonication [8] with ultrafiltration. Ultrasound irradiation has been shown to continuously refresh the membrane–foulant interface, while the application of an electric field induces protein denaturation, promoting the formation of larger flocs and reducing irreversible fouling. Pre-treatment methods, such as pre-chlorination and pre-ozonation of protein-containing feed streams [9,10,11], have also been employed. These pre-treatments weaken protein–membrane and intra-protein interactions, disrupting the adsorption–desorption cycle and ultimately minimizing deposition. Additionally, aluminum coagulant pre-treatment [12,13] has been utilized to alter the hydrophobicity of bulk proteins, thereby weakening interactions within the fouling layer. Membrane modification represents another effective strategy to address fouling. Techniques such as plasma grafting [14] and the incorporation of polyethylene glycol additives [15] have been applied to enhance membrane hydrophilicity, reducing the interaction between hydrophobic proteins and the membrane. Furthermore, electrochemical enhancement through the introduction of negative charges to the membrane surface has been reported to suppress foulant adhesion via electrostatic repulsion, improving fouling resistance [16,17].

The incorporation of photocatalysts into the membrane matrix, forming a so-called photocatalytic membrane, is another promising modification strategy to address membrane fouling. Photocatalysis has been viewed as a sustainable environmental technology to mineralize target pollutant in wastewater [18,19,20]. Photocatalytic membranes—whether coated, blended, or free-standing—facilitate photocatalysis to degrade the foulants under light irradiation while maintaining the membrane’s selectivity and permeability. Yang et al. [21] blended a mesoporous graphitic carbon nitride photocatalyst into a poly(vinylidene fluoride) membrane (PVDF). The resulting photocatalytic membrane demonstrated the capability to degrade approximately 97% of cefotaxime under sunlight irradiation. Similarly, Wan et al. [22] developed a photocatalytic membrane by coating a bismuth molybdate graphitic carbon nitride composite photocatalyst on a PVDF substrate. Their study revealed that the membrane demonstrated an improved degradation of tetracycline hydrochloride to 67.85%, while achieving an 85% flux recovery ratio within 30 min of visible light irradiation, using bovine serum albumin (BSA) and sodium alginate as model foulants.

In a previous work [23], a ternary tungsten trioxide (WO_3_)/carbon nanotube (CNT)/zinc oxide (ZnO) photocatalyst was developed to resolve the inherent limitation of a ZnO photocatalyst in the photocatalytic degradation of methylene blue (MB) and BSA. The lower band gap of WO_3_, combined with the effective charge transfer facilitated by the CNTs and the formation of a Z-scheme heterojunction in the ternary photocatalyst, significantly enhanced the photocatalytic degradation of MB and BSA. Building on this, the present study focuses on the fabrication of a free-standing photocatalytic membrane, referred to as the ternary WO_3_/CNT/ZnO–chitosan composite photocatalytic membrane using the ternary photocatalyst to mitigate protein fouling in the membrane. The ternary composite photocatalytic membrane combines the self-assembly properties of CNT in the ternary photocatalyst with chitosan as a binder to construct a photocatalytically active porous membrane layer with selective permeation properties [24]. The proposed synthesis approach eliminates the need for a supporting layer, enabling the formation of a thinner membrane. The membrane’s photocatalytic activity, permeation performance, and antifouling properties were evaluated using BSA as a model foulant.

## 2. Material and Methodology

### 2.1. Materials

WO_3_ and polyvinylpyrrolidone (PVP) were purchased from Alfa Aesar (Stoughton, MA, USA) while CNT with the dimensions of 30–50 μm in length and 8–15 nm in diameter was supplied by Nova Scientific (Selangor, Malaysia). In addition, zinc nitrate hexahydrate, 30% ammonium hydroxide solution and polyethylene glycol 2000 (PEG) were obtained from Systerm (Selangor, Malaysia), Synerlab (Selangor, Malaysia) and Merck (Selangor, Malaysia), respectively. Low (50–190 kDa), medium (190–310 kDa) and high (310–375 kDa) molecular weight chitosan was bought from Sigma-Aldrich (Selangor, Malaysia). Lastly, ethanol, BSA and acetic acid were procured from Gene Chemicals (Selangor, Malaysia), Sigma-Aldrich (Malaysia) and HmbG Chemicals (Selangor, Malaysia), respectively.

### 2.2. Synthesis of Ternary Composite Photocatalytic Membrane

**Figure 1:**
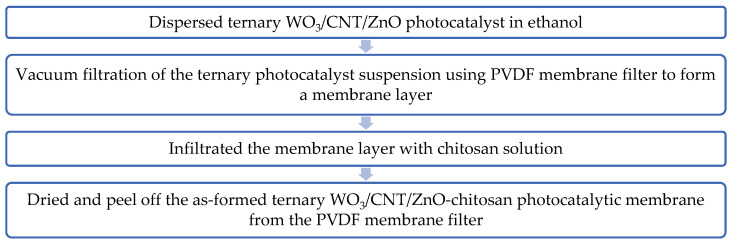
Flowchart of the ternary WO_3_/CNT/ZnO–chitosan composite photocatalytic membrane preparation process.

The ternary WO_3_/CNT/ZnO photocatalyst was synthesized following the procedure reported in a previous study [23]. The ternary WO_3_/CNT/ZnO photocatalyst was then casted into a membrane layer through a wet processing technique. The ternary photocatalyst was dispersed in 150 mL of ethanol and sonicated for 90 min. The suspensions were then subjected to a vacuum filtration with a 0.22 μm PVDF membrane filter to form a thin membrane layer. The membrane layer formed was rinsed with distilled water and followed by infiltration with 1 g/L of chitosan solution to form a so-called ternary WO_3_/CNT/ZnO–chitosan composite photocatalytic membrane. Chitosan of low, medium and high molecular weight was used in the infiltration process. The as-formed ternary WO_3_/CNT/ZnO–chitosan composite photocatalytic membranes were dried at room temperature overnight before being peeled off from the PVDF membrane filter. A flowchart depicting the preparation process of the ternary WO_3_/CNT/ZnO–chitosan composite photocatalytic membrane is shown in Figure 1. The photocatalytic membranes were prepared using five different loadings of the ternary photocatalyst, i.e., 0.08 g (M1), 0.10 g (M2), 0.12 g (M3), 0.14 g (M4), and 0.16 g (M5).

### 2.3. Characterization of Ternary Composite Photocatalytic Membrane

The tensile mechanical properties of the ternary composite photocatalytic membrane were studied using a universal tensile tester (Tinius Olsen-H10KS, Horsham, PA, USA). The ternary composite photocatalytic membrane was cut into a dumbbell shape with a gauge length of 2 cm and the thickness was measured by a thickness gauge. The running speed of the tensile tester was set at 0.5 mm/min. The surface morphology, cross-section and elemental analysis of the ternary composite photocatalytic membrane were visualized using field emission scanning electron microscopy (FESEM, JEOL JSM-6701F, Tokyo, Japan) equipped with the energy dispersive X-ray (EDX) function. The ternary composite photocatalytic membranes were fractured in liquid nitrogen and coated with silver prior to the characterization. The chemical structure of the ternary composite photocatalytic membrane was evaluated using Fourier transform infrared (FTIR, Perkin Elmer Spectrum Two, Waltham, MA, USA) with a spectral range from 500 cm^−1^ to 4000 cm^−1^.

The porosity of the ternary composite photocatalytic membrane was assessed via a gravimetric method in which the membrane was immersed in distillate water for at least 12 h to ensure that its pores were fully filled with water. The average porosity, *p*, was subsequently calculated using the following equation [25]:

(1) $$p=\frac{w_{2}-w_{1}}{A\times l\times \rho_{w}}\times 100\%$$

where *w*_1_ (kg) is the weight of the dry membrane, *w*_2_ (kg) is the weight of the wet membrane, *A* (m^2^) is the effective surface area of the membrane, *ℓ* (m) is the thickness of the membrane and *ρ_w_* (kg/m^3^) is the density of water (1000 kg/m^3^).

In addition, the mean pore radius (*r_m_*) of the ternary composite photocatalytic membrane was determined by the Guerout–Elford–Ferry relation as shown below [25]:

(2) $$r_{m}=\sqrt{\frac{(2.9-1.75p)\times 8\eta lQ}{p\times A\times ∆P}}$$

where *η* (Pa s) represents the water viscosity, which is 8.9 × 10^−4^ Pa s, *Q* (m^3^/s) is the volumetric flow rate of water and Δ*P* (Pa) refers to the transmembrane pressure.

### 2.4. Photocatalytic Activity, Permeation and Antifouling Properties of the Ternary Composite Photocatalytic Membranes

The photocatalytic activity of the ternary composite photocatalytic membrane was evaluated by its ability to degrade BSA at a specific time interval under light irradiation. The ternary composite photocatalytic membrane was fixed in a Petri dish filled with 1000 mg/L of BSA solution and kept in the dark for 30 min to reach the adsorption equilibrium, followed by 2 h of natural sunlight irradiation. The concentration of BSA solution was measured using a UV–visible spectrophotometer (WPA Lightwave II, Southampton, UK) in the wavelength of 292 nm [26]. The photocatalytic activity of the ternary composite photocatalytic membrane was assessed based on Equation (3) [27].

(3) $$D_{e}=\frac{C_{0}-C}{C_{0}}\times 100\%$$

where *D_e_* (%) denotes the degradation efficiency, *C*_0_ (mg/L) denotes the initial concentration of BSA solution and *C* (mg/L) denotes the residual concentration of BSA solution.

Permeation characteristic of ternary composite photocatalytic membranes was carried out using a dead-end membrane filtration system with an effective surface area of 0.0011 m^2^. The feed solution consisted of 1000 mg/L of BSA solution, which was to simulate the typical protein concentration found in real wastewater. The operating pressure applied was kept constant at 0.3 MPa and the contact time was set as 30 min. The permeation flux (*J*) of the membrane was determined using the following equation:

(4) $$J=\frac{V}{A\times t}$$

where *V* (L) is the total volume of permeate collected and *t* (h) is the permeation time.

The concentrations of permeate and feed solutions were measured by a UV–visible spectrophotometer in the wavelength of 292 nm and the rejection property of the membrane (*R*) was calculated using the equation below:

(5) $$R=\frac{{C_{f}-C}_{p}}{C_{f}}\times 100\%$$

where *C_p_* (mg/L) and *C_f_* (mg/L) represent the concentration of BSA in the permeate and feed solutions.

The antifouling performance of the ternary composite photocatalytic membrane was assessed using BSA as model foulant. Firstly, the ternary composite photocatalytic membrane was permeated with distilled water and the permeation flux (*J_w_*_1_) was recorded. Subsequently, the membrane was subjected to permeation with 1000 mg/L BSA solution. The fouled membrane was exposed to sunlight irradiation for 30 min to activate the photocatalysis to degrade the accumulated foulant on the membrane. A control study was conducted in which the fouled membrane was not exposed to sunlight irradiation. Lastly, the ternary composite photocatalytic membrane was permeated with distilled water again and the permeation flux (*J_w_*_2_) was recorded. The antifouling property of the membrane was analyzed based on the flux recovery ratio (*R_fr_*) and irreversible fouling ratio (*R_if_*) [28], as shown below:

(6) $$R_{fr}=\frac{J_{w2}}{J_{w1}}\times 100\%$$

(7) $$R_{if}=\frac{J_{w1}-J_{w2}}{J_{w1}}\times 100\%$$

The reusability test was carried out to assess the stability performance of the ternary composite photocatalytic membrane. The membrane was permeated with BSA solution, after which the permeation flux and rejection were recorded. Subsequently, the membrane was exposed to sunlight irradiation for 30 min before undergoing a second cycle of BSA solution permeation. The procedures were repeated for four consecutive cycles. A control experiment was performed by skipping the sunlight irradiation procedure.

## 3. Results and Discussion

### 3.1. Mechanical Properties of the Ternary Composite Photocatalytic Membrane

**Figure 2:**
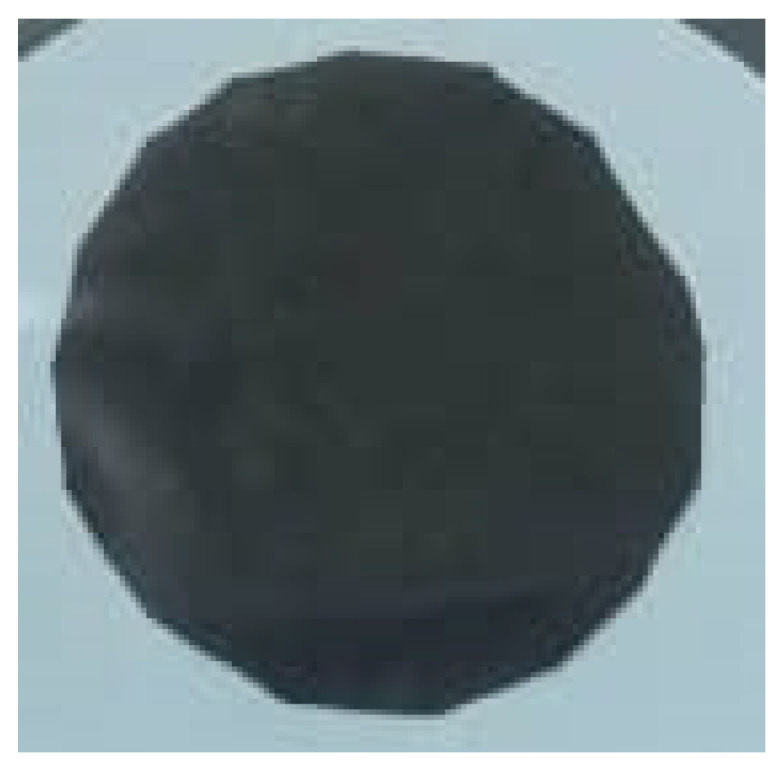
Ternary WO_3_/CNT/ZnO–chitosan composite photocatalytic membrane (dark layer) on a PVDF membrane filter (white layer).

The ternary WO_3_/CNT/ZnO composite photocatalytic membrane was successfully formed via a wet processing technique. Yet, the physical stability of the ternary composite photocatalytic membrane was relatively weak; hence, it was infiltrated with chitosan to enhance its structural integrity. Figure 2 shows the as-formed ternary WO_3_/CNT/ZnO composite photocatalytic membrane on a PVDF membrane filter.

**Figure 3:**
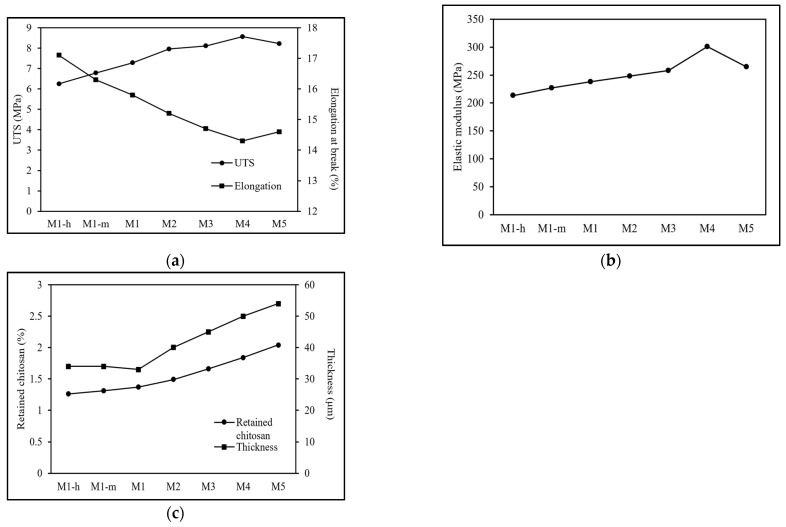
(**a**) UTS and elongation at break, (**b**) elastic modulus, and (**c**) retained chitosan and thickness of ternary composite photocatalytic membrane with different types of infiltrated chitosan and loadings of ternary photocatalyst.

This can be attributed to the presence of chitosan that serves as a binding agent to connect the individual photocatalysts. The interaction of chitosan and ternary photocatalyst can be established through formation of hydrogen bonding between the functional moieties of chitosan (-NH_2_ and -OH) and the -OH functional moiety of the CNT in the ternary photocatalyst, which allows the chitosan to distribute the stress within the ternary composite photocatalytic membrane [29]. The influence of chitosan’s molecular weight was carried out by infiltrating the ternary composite photocatalytic membrane with high (M1-h), medium (M1-m) and low (M1) molecular weight chitosan. The outcome, as illustrated in Figure 3, demonstrated that the ultimate tensile strength (UTS) and elastic modulus were gradually increased when infiltrated with lower molecular weight chitosan, while the elongation at break of the ternary composite photocatalytic membrane was slightly decreased. Chitosan with different molecular weights contains varying lengths of molecular chains that result in different levels of viscosity [30,31]. Higher molecular weight chitosan exhibited increased viscosity as a result of its longer molecular chain, thereby limiting the penetration of chitosan into the ternary photocatalyst layer. In contrast, lower molecular weight chitosan penetrates more easily, resulting in higher retention within the membrane, as shown in Figure 3c. The high retention of lower molecular weight chitosan allows the M1 ternary composite photocatalytic membrane to attain the highest UTS (7.28 MPa) and elastic modulus (238 MPa) but at the expense of elongation at break (15.8%). Meanwhile, it is noticed that the thickness of the M1-h, M1-m, and M1 photocatalytic membranes remained nearly unchanged, indicating that the different molecular weights of chitosan do not significantly contribute to layer formation on the photocatalytic membrane. Anticipating the highest UTS and elastic modulus exhibited in the M1 photocatalytic membrane, the low molecular weight chitosan (M1) was then applied in the subsequent study to investigate the influence of the ternary photocatalyst’s loading on the mechanical properties of the formed ternary composite photocatalytic membrane.

The UTS and elastic modulus increased from 7.28 MPa to 8.56 MPa and from 238 MPa to 301 MPa, respectively, with increasing loading of the ternary photocatalyst from 0.08 g (M1) to 0.14 g (M4). The formation of the ternary photocatalyst layer can be mainly ascribed to the entanglement of CNT within the ternary photocatalyst. Thus, increasing the loading of the ternary photocatalyst signifies a greater presence of CNT within the ternary composite photocatalytic membrane, leading to heightened entanglement and consequently yielding better mechanical stability. Additionally, the increasing ternary photocatalyst’s loading also led to an increase in the thickness of the ternary composite photocatalytic membrane layer, which enables more chitosan to be entrapped or retained within the ternary composite photocatalytic membrane. This would intensify the interaction of the chitosan and ternary photocatalyst to withstand a higher load [32]. However, the UTS and elastic modulus of the ternary composite photocatalytic membrane decreased when the loading of the ternary photocatalyst was increased to 0.16 g (M5). A higher ternary photocatalyst loading resulted in the formation of large agglomeration, which reduced the contact area between the chitosan and ternary photocatalyst, eventually leading to an uneven distribution of chitosan and diminishing of the hydrogen bond formed within the ternary composite photocatalytic membrane, although more chitosan was retained in the ternary composite photocatalytic membrane [33]. The uneven distribution of chitosan ultimately led to the de-bonding phenomenon and impeded the stress transfer in the membrane, thereby reducing its tensile strength [34,35].

### 3.2. Morphology Analysis of Ternary Composite Photocatalytic Membrane

**Figure 4:**
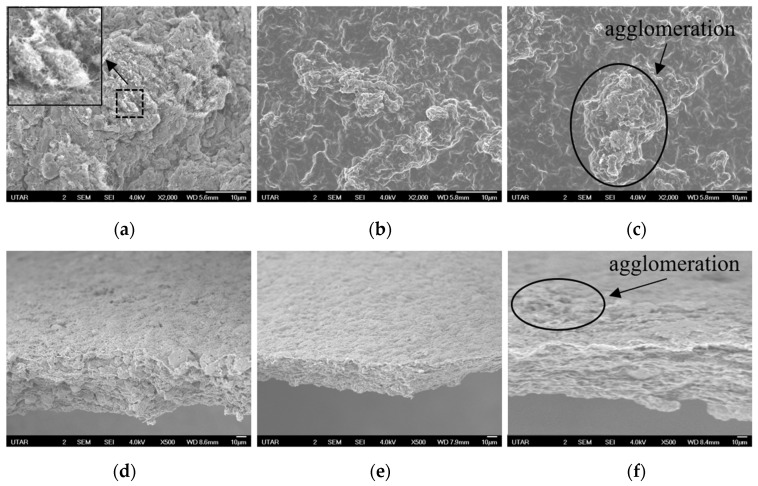
FESEM images of surface morphology for (**a**) M1 photocatalytic membrane before infiltration with chitosan. The inset figure shows higher magnification image, (**b**) M1 photocatalytic membrane and (**c**) M5 photocatalytic membrane. Cross-sectional view of the ternary composite photocatalytic membrane, (**d**) M1 photocatalytic membrane before infiltration with chitosan, (**e**) M1 photocatalytic membrane and (**f**) M5 photocatalytic membrane.

The morphology of the ternary composite photocatalytic membrane before and after infiltration with chitosan are illustrated in Figure 4. The surface morphology of the ternary composite photocatalytic membrane before infiltration with chitosan, as depicted in Figure 4a, showed the entanglement of CNT thread in the ternary WO_3_/CNT/ZnO photocatalyst which was responsible for the assembly of membrane layer and formation of interstitial porous structure. For the ternary composite photocatalytic membrane after infiltrated with chitosan, as displayed in Figure 4b,c, it can be observed that the ternary photocatalyst was coated with the chitosan. A larger agglomeration was visible in Figure 4c for M5 photocatalytic membrane as a result of the high loading of ternary photocatalyst which weaken the binding effect from the chitosan. In terms of the cross-sectional view of the M1 photocatalytic membrane before and after infiltration with chitosan, as shown in Figure 4d–f, respectively, the thickness of the M1 photocatalytic membrane was found to be decreased after infiltration with chitosan. The phenomenon indicates a good interaction between chitosan and the ternary photocatalyst where the contact of chitosan with the ternary photocatalyst led to the shrinkage of the ternary composite photocatalytic membrane into a compact structure upon infiltration.

### 3.3. Elemental Composition Analysis of Ternary Composite Photocatalytic Membrane

**Figure 5:**
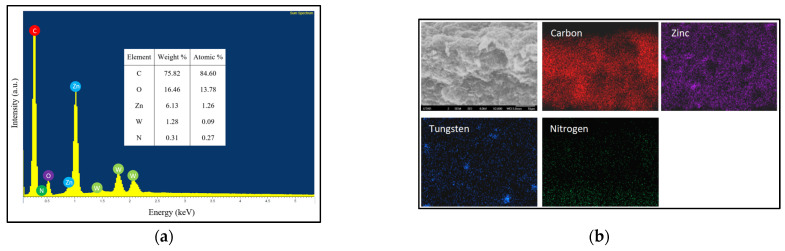
(**a**) EDX spectrum, and (**b**) EDX mapping of M1 photocatalytic membrane.

The EDX spectrum of the M1 photocatalytic membrane is shown in Figure 5a. Several types of elements including C, O, Zn and W were detected, which could indicate the existence of CNT, ZnO and WO_3_. In addition, the nitrogen (N) element was detected, which is associated with the chitosan trapped within the ternary composite photocatalytic membrane. The elements detected were well-distributed across the ternary composite photocatalytic membrane, as shown in the EDX mapping in Figure 5b. The findings from the FESEM and EDX further confirmed the retention of chitosan after the infiltration process, which presented advantages in connecting the individual ternary photocatalyst.

### 3.4. FTIR Analysis of Ternary Composite Photocatalytic Membrane

**Figure 6:**
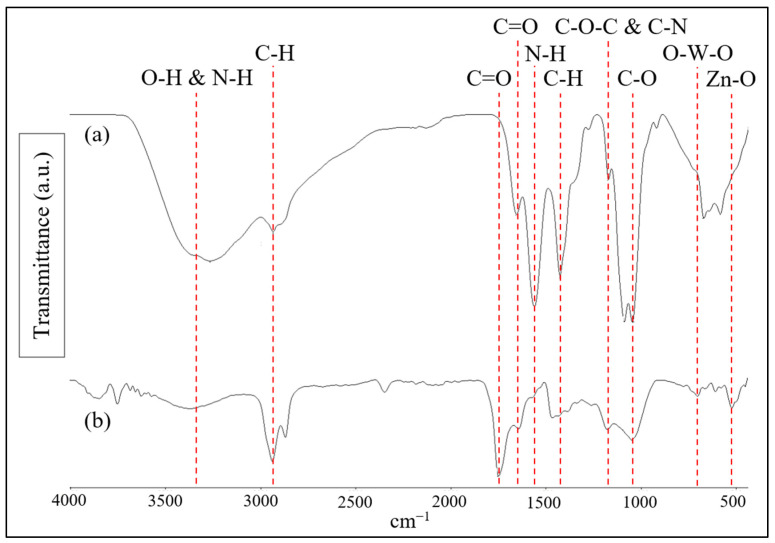
FTIR spectra of (a) low molecular weight chitosan and (b) M1 photocatalytic membrane.

The FTIR spectra of low molecular weight chitosan and the M1 ternary composite photocatalytic membranes are presented in Figure 6. The low molecular weight chitosan exhibited a characteristic peak at approximately 3330 cm^−1^, which was ascribed to the stretching of O-H and N-H bonds [36]. In addition, the absorption peak at approximately 2920 cm^−1^ can be associated with the C-H stretching and the peaks at 1640 cm^−1^ and 1550 cm^−1^ can be assigned to the C=O group (amide I) and N-H bending (amide II) of amide in chitosan [37,38]. Additionally, the absorption peaks at 1400 cm^−1^, 1150 cm^−1^ and 1030 cm^−1^ were also observed, which correspond to the C-H bending, C-O-C and C-N stretching, and skeletal vibration of C-O stretching, respectively, in chitosan [39].

For the M1 photocatalytic membrane, peaks corresponding to the C=O group, O-W-O stretching, and Zn-O stretching were observed at 1730 cm^−1^, 750 cm^−1^, and 510 cm^−1^, respectively. These observations align with previously reported FTIR data [23], further confirming the presence of CNT, WO_3_, and ZnO within the membrane. In addition, the functional groups associated with chitosan, including the amide I peak and C-O-C peak, were detected at around 1640 cm^−1^ and 1150 cm^−1^, respectively. These findings indicate that chitosan is incorporated within the ternary composite photocatalytic membrane. Moreover, the presence of the hydroxyl and amide group in the ternary composite photocatalytic membrane facilitates the formation of hydrogen bonds. As mentioned above, this contributes to enhancing the structure stability of the membrane [29].

### 3.5. Porosity and Pore Radius of Ternary Composite Photocatalytic Membrane

**Figure 7:**
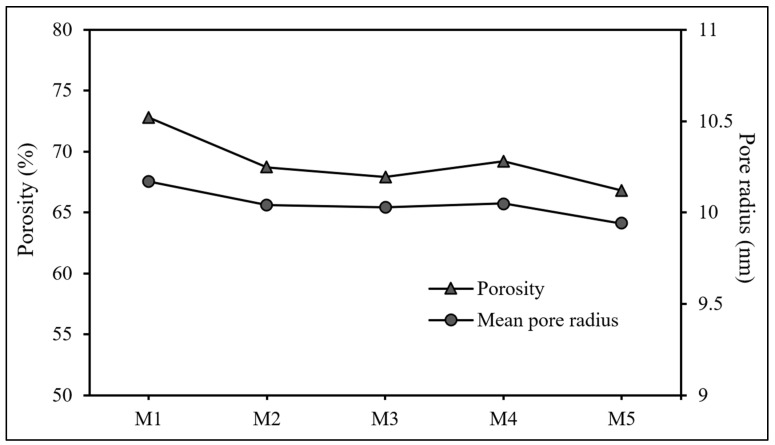
Average porosity and average pore radius of ternary composite photocatalytic membranes.

The average porosity and mean pore radius of the ternary composite photocatalytic membrane are depicted in Figure 7. The average porosity of the ternary composite photocatalytic membranes steadily decreased from 72.8% to 66.8% when the loading of the ternary photocatalyst in the membrane was increased from 0.08 g (M1) to 0.16 g (M5). Meanwhile, the mean pore radius of the ternary composite photocatalytic membranes exhibited a trend consistent with that observed for porosity. A slight but continuous decrease in the mean pore radius of the ternary composite photocatalytic membranes from 10.17 nm (M1) to 9.94 nm (M5) was observed with increasing loading of the ternary photocatalyst. It can be inferred that the increased ternary photocatalyst loading increases the packing density of the ternary composite photocatalytic membrane, leading to a reduction in void volumes within the membrane structure. In addition, as reported in Figure 3c, the chitosan trapped within the ternary composite photocatalytic membrane was found to be increased with increasing loading of the ternary photocatalyst. As a result, the interstitial spaces could be filled with chitosan and reduce the void volumes within the ternary composite photocatalytic membrane. In addition, the infiltrated chitosan inside the ternary composite photocatalytic membrane could shrink during the drying process, which would further decrease the mean pore size of the ternary composite photocatalytic membrane.

### 3.6. Photodegradation of Bovine Serum Albumin by Ternary Composite Photocatalytic Membrane

**Figure 8:**
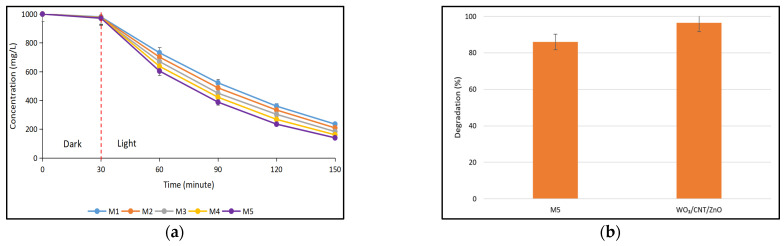
(**a**) Photodegradation of BSA (1000 mg/L) using ternary composite photocatalytic membranes. (**b**) Comparison of the photodegradation of BSA between M5 photocatalytic membrane and ternary composite photocatalyst.

Figure 8a demonstrates the photocatalytic activity of the ternary composite photocatalytic membranes in degrading the BSA. All the ternary composite photocatalytic membranes were able to degrade the BSA under light irradiation for 120 min. The photodegradation of BSA was found to be increased with increasing ternary photocatalyst loading in the membrane wherein the M5 membrane attained the highest BSA degradation (86%). The increased degradation with increasing loading of the photocatalyst in the membrane was indeed a commonly observed trend. A higher loading of photocatalyst in the ternary composite photocatalytic membrane provides an increased surface area and more active sites, facilitating the generation of more radicals upon receiving sufficient energy from sunlight. These radicals actively participate in the photocatalytic reaction, targeting BSA molecules [40,41]. The incorporation of CNT and WO_3_ also aids in promoting the photocatalytic activity of the ternary composite photocatalytic membrane toward the degradation of BSA by providing effective charge separation and charge transfer and improving the sunlight absorption capability [23,42,43]. The photodegradation of BSA can be attributed to the generation of radical species by the ternary photocatalyst during sunlight irradiation, which break the peptide chains of BSA into smaller molecules, and ultimately degrade them into CO_2_ and H_2_O [44,45,46].

The photocatalytic performance of the ternary photocatalyst was evaluated in both particle and membrane forms, with the results presented in Figure 8b. The loading of the ternary photocatalyst in the particle and membrane forms was kept constant at 0.16 g. The ternary photocatalyst in particle form obtained 96% BSA degradation, while the membrane form (M5 photocatalytic membrane) attained 86% BSA degradation. This outcome displayed that the photocatalytic performance of the ternary photocatalyst was slightly attenuated after being assembled into membrane form. However, it still remained comparable in terms of its efficiency in BSA degradation. It is interesting to note that the infiltration of chitosan did not drastically reduce the photodegradation performance of the ternary composite photocatalytic membrane.

### 3.7. Permeability and Rejection Analysis of Ternary Composite Photocatalytic Membrane

**Figure 9:**
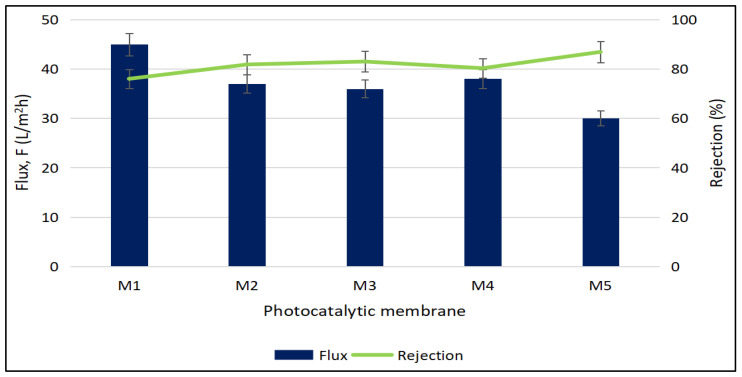
Permeation and rejection of ternary composite photocatalytic membrane on BSA filtration.

The influence of the ternary photocatalyst’s loading on the permeation property of the ternary composite photocatalytic membranes was evaluated in terms of permeation flux and rejection toward BSA solution and the result is illustrated in Figure 9. The permeation flux of the ternary composite photocatalytic membrane on BSA solution overall exhibited a marginal decreasing trend from 45 L/m^2^·h to 30 L/m^2^·h when the loading of the ternary photocatalyst in the membrane was increased. The permeation flux of the ternary composite photocatalytic membrane primarily relied on the porous structure of the ternary composite photocatalytic membrane. The observed decrease in the permeation flux is due to the decrease in the average porosity and mean pore radius as reported in Figure 6, since the reduced average porosity and smaller mean pore radius impose higher resistance to fluid flow.

On the other hand, the rejection efficiency of the ternary composite photocatalytic membrane marginally increased from 76% to 86.9% with higher loading of the ternary photocatalyst. BSA has a molecular weight of 66,800 g/mol [47], which makes it prone to rejection during permeation in the ternary composite photocatalytic membrane, with some being captured and retained within the membrane. Moreover, the smaller pore size with increasing loading of the photocatalyst in the ternary composite photocatalytic membrane favored the entrapment of BSA molecules. This led to an increase in flow resistance and a reduction in the permeation flux of the ternary composite photocatalytic membrane [48,49], hence providing a higher rejection of the ternary composite photocatalytic membrane on BSA solution at the expense of permeation flux. Overall, the ternary composite photocatalytic membrane demonstrated a relatively high rejection rate toward the BSA.

### 3.8. Antifouling Analysis of Ternary Composite Photocatalytic Membrane

**Figure 10:**
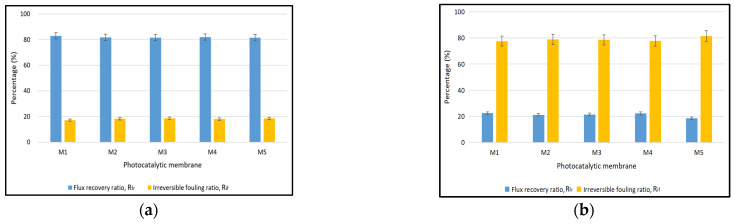
Antifouling properties of the ternary composite photocatalytic membrane (**a**) with photocatalytic treatment (treated membrane with sunlight irradiation), and (**b**) without photocatalytic treatment (untreated membrane).

The antifouling properties of the ternary composite photocatalytic membrane were analysed by assessing the *R_fr_* and *R_if_*, as demonstrated in Figure 10. The *R_fr_* of all ternary composite photocatalytic membranes was around 82% and the *R_if_* was about 18% as displayed in Figure 10a. A control experiment was carried out using the ternary composite photocatalytic membrane in which the photocatalytic treatment with sunlight irradiation was omitted after the permeation of BSA solution, and the result is shown in Figure 10b. A relatively low *R_fr_* of approximately 20% was observed for the ternary composite photocatalytic membrane in the control experiment. In addition, the *R_if_* of the ternary composite photocatalytic membrane in the control experiment was approximately 80%, which was remarkably high. The results obtained from the control experiment clearly demonstrated severe fouling in the ternary composite photocatalytic membrane in the absence of photocatalytic treatment. As mentioned earlier, the ternary composite photocatalytic membrane showed high rejection toward the BSA; hence, there is a high chance that the rejected BSA molecules are deposited in the membrane and act as a foulant to obstruct the porous pathway, thus reducing the permeability of the membrane. As compared to the result obtained in Figure 10, *R_fr_* was substantially improved from 20% to 82% while *R_if_* was significantly reduced from 80% to 18% when the ternary composite photocatalytic membrane was subjected to photocatalytic treatment. Once again, this reveals that the presence of the ternary composite photocatalyst in the membrane enabled the photocatalysis mechanism to degrade and eliminate the foulants (BSA) when exposed to light irradiation. Consequently, it helps mitigate fouling caused by the deposition and accumulation of foulants, thereby enhancing the flux recovery of the ternary composite photocatalytic membrane.

### 3.9. Reusability Analysis of Ternary Composite Photocatalytic Membrane

**Figure 11:**
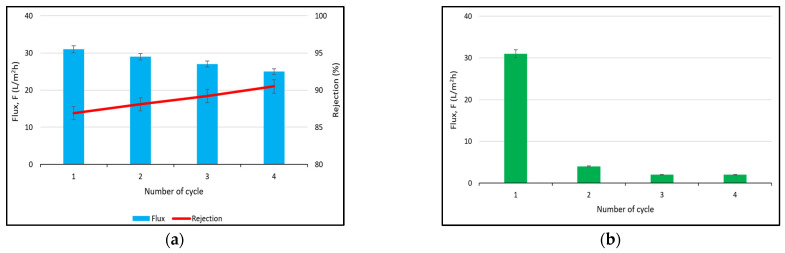
Reusability analysis of ternary composite photocatalytic membrane on BSA filtration (**a**) with and (**b**) without the photocatalytic treatment.

Reusability analysis was conducted in order to measure the persistent permeation performance of the ternary composite photocatalytic membrane. The M5 photocatalytic membrane was utilized in this analysis since it exhibited higher rejection toward BSA solution. The permeation flux and rejection of the ternary composite photocatalytic membrane on BSA solution are displayed in Figure 11. The permeation flux of the ternary composite photocatalytic membrane on BSA solution suffered from a small decrease from 31 L/m^2^·h to 25 L/m^2^·h while the BSA rejection was slightly increased from 86.9% to 90.5% after four consecutive cycles, as shown in Figure 11a. This could likely be due to the mild fouling formed in the membrane. It is worth noting that the reusability of the ternary composite photocatalytic membrane in the BSA solution strongly depends on its antifouling properties. The reusability of the ternary composite photocatalytic membrane was hindered after one cycle when the membrane was not subjected to photocatalytic treatment. The membrane was severely blocked by the BSA after one cycle as displayed in Figure 11b. The finding reveals the importance of the antifouling behaviour in the ternary composite photocatalytic membrane to maintain the reusability of the membrane.

## 4. Conclusions

The ternary WO_3_/CNT/ZnO–chitosan composite photocatalytic membrane was successfully formed via the wet processing method and infiltration with chitosan. Notably, the infiltration with low molecular weight chitosan emerged as a promising binding agent to the ternary composite photocatalytic membrane by providing relatively high mechanical stability. The surface morphology of the ternary composite photocatalytic membrane using FESEM found that the photocatalyst was assembled into a membrane layer due to the entanglement of CNT and the infiltration of chitosan facilitated the contact between the ternary photocatalyst. The increased loading of ternary photocatalyst in the ternary composite photocatalytic membrane up to 0.14 g (M4) improved the tensile strength and elastic modulus of the membrane while both the tensile strength and elastic modulus decreased beyond the 0.14 g loading of the ternary photocatalyst as a result of agglomeration. The photocatalytic performance of the ternary composite photocatalytic membrane in BSA degradation was found to be increased with incremental loading of the ternary photocatalyst and the highest BSA degradation (86%) was attained in the M5 photocatalytic membrane. The permeation flux of the ternary composite photocatalytic membrane toward 1000 mg/L of BSA solution steadily declined with increasing loading of the ternary photocatalyst while the rejection toward BSA was increased, which can be associated with the decreased average porosity and mean pore radius. The ternary composite photocatalytic membrane demonstrated reasonably good antifouling behavior with an *R_fr_* of 82% and an *R_if_* of 18%. The antifouling property of the ternary composite photocatalytic membrane is important in maintaining the reusability of the membrane.

## Data Availability

Raw data that support the finding of this study are available from the corresponding author upon request.

## References

[1] Cassano A., Basile A. (2011). Membranes for industrial microfiltration and ultrafiltration. Elsevier eBooks.

[2] Conidi C., Donato L., Cassano A. (2023). Membrane processes in food and pharmaceutical industries. Elsevier eBooks.

[3] Corbatón-Báguena M., Álvarez-Blanco S., Vincent-Vela M. (2015). Fouling mechanisms of ultrafiltration membranes fouled with whey model solutions. Desalination.

[4] Gavazzi-April C., Benoit S., Doyen A., Britten M., Pouliot Y. (2018). Preparation of milk protein concentrates by ultrafiltration and continuous diafiltration: Effect of process design on overall efficiency. J. Dairy Sci..

[5] Xu G., Tobin J., Amani H., Subhir S., O’Donnell C., O’Shea N. (2025). Potential of acoustic sensors for real-time monitoring of physicochemical properties of milk protein concentrate during ultrafiltration. J. Food Eng..

[6] Tanudjaja H., Anantharaman A., Ng A., Ma Y., Tanis-Kanbur M., Zydney A., Chew J. (2022). A review of membrane fouling by proteins in ultrafiltration and microfiltration. J. Water Proc. Eng..

[7] Song W., Su Y., Chen X., Ding L., Wan Y. (2010). Rapid concentration of protein solution by a crossflow electro-ultrafiltration process. Sep. Purif. Technol..

[8] Koh L., Nguyen H., Chandrapala J., Zisu B., Ashokkumar M., Kentish S. (2014). The use of ultrasonic feed pre-treatment to reduce membrane fouling in whey ultrafiltration. J. Membr. Sci..

[9] Miao R., Feng Y., Wang Y., Wang P., Li P., Li X., Wang L. (2021). Exploring the influence mechanism of ozonation on protein fouling of ultrafiltration membranes as a result of the interfacial interaction of foulants at the membrane surface. Sci. Total Environ..

[10] Miao R., Zhou Y., Wang P., Lu W., Li P., Li X., Wang L. (2021). A comparison of effect mechanisms of chlorination and ozonation on the interfacial forces of protein at membrane surfaces and the implications for membrane fouling control. J. Membr. Sci..

[11] Song J., Zhang Z., Tang S., Tan Y., Zhang X. (2018). Does pre-ozonation or in-situ ozonation really mitigate the protein-based ceramic membrane fouling in the integrated process of ozonation coupled with ceramic membrane filtration?. J. Membr. Sci..

[12] Zhou M., Meng F. (2016). Aluminum-induced changes in properties and fouling propensity of DOM solutions revealed by UV–vis absorbance spectral parameters. Water Res..

[13] Tang C., Peleato N., Bérubé P., Andrews R. (2019). Impact of low coagulant dosages on protein fouling of ultrafiltration membranes. J. Water Proc. Eng..

[14] Khongnakorn W., Bootluck W., Jutaporn P. (2020). Surface modification of FO membrane by plasma-grafting polymerization to minimize protein fouling. J. Water Proc. Eng..

[15] Ma Y., Shi F., Ma J., Wu M., Zhang J., Gao C. (2011). Effect of PEG additive on the morphology and performance of polysulfone ultrafiltration membranes. Desalination.

[16] Valiño V., Román M., Ibañez R., Ortiz I. (2014). Improved separation of bovine serum albumin and lactoferrin mixtures using charged ultrafiltration membranes. Sep. Purif. Technol..

[17] Fan X., Liu Y., Wang X., Quan X., Chen S. (2019). Improvement of Antifouling and Antimicrobial Abilities on Silver–Carbon Nanotube Based Membranes under Electrochemical Assistance. Environ. Sci. Technol..

[18] Nasrollahi N., Ghalamchi L., Vatanpour V., Khataee A. (2021). Photocatalytic-membrane technology: A critical review for membrane fouling mitigation. J. Ind. Eng. Chem..

[19] Ganguly P., Byrne C., Breen A., Pillai S. (2018). Antimicrobial activity of photocatalysts: Fundamentals, mechanisms, kinetics and recent advances. Appl. Catal. B Environ..

[20] Goktas S., Goktas A. (2021). A comparative study on recent progress in efficient ZnO based nanocomposite and heterojunction photocatalysts: A Review. J. Alloys Compd..

[21] Yang F., Ding G., Wang J., Liang Z., Gao B., Dou M., Xu C., Li S. (2020). Self-cleaning, antimicrobial, and antifouling membrane via integrating mesoporous graphitic carbon nitride into polyvinylidene fluoride. J. Membr. Sci..

[22] Wan J., Huang J., Yu H., Liu L., Shi Y., Liu C. (2021). Fabrication of self-assembled 0D-2D Bi_2_MoO_6_-g-C_3_N_4_ photocatalytic composite membrane based on PDA intermediate coating with visible light self-cleaning performance. J. Colloid Interface Sci..

[23] Chong W., Lam S., Don T., Ong Y. (2023). Improved photocatalytic activity of zinc oxide through the formation of novel ternary tungsten trioxide/carbon nanotube/zinc oxide composite photocatalyst. Mater. Sci. Eng. B.

[24] Elnabawy E., Elsherbiny I., Abdelsamad A., Anis B., Hassan A., Ulbricht M., Khalil A. (2020). Tailored CNTs buckypaper membranes for the removal of humic acid and separation of oil-in-water emulsions. Membranes.

[25] Rabiee H., Vatanpour V., Farahani M., Zarrabi H. (2015). Improvement in flux and antifouling properties of PVC ultrafiltration membranes by incorporation of zinc oxide (ZnO) nanoparticles. Sep. Purif. Technol..

[26] Buddanavar A., Nandibewoor S. (2017). Multi-spectroscopic characterization of bovine serum albumin upon interaction with atomoxetine. J. Pharm. Anal..

[27] Roozban N., Abbasi S., Ghazizadeh M. (2017). The experimental and statistical investigation of the photo degradation of methyl orange using modified MWCNTs with different amount of ZnO nanoparticles. J. Mater. Sci. Mater. Electron..

[28] Zhang Q., Liu Y., Su Y., Zhang R., Fan L., Liu Y., Ma T., Jiang Z. (2016). Fabrication and characterization of antifouling carbon nanotube/polyethersulfone ultrafiltration membranes. RSC Adv..

[29] Alosime E., Alshahrani A., Nghiem L., in het Panhuis M. (2019). The preparation and characterization of Buckypaper made from carbon nanotubes impregnated with chitosan. Polym. Compos..

[30] Zhang H., Li Y., Zhang X., Liu B., Zhao H., Chen D. (2016). Directly determining the molecular weight of Chitosan with Atomic Force Microscopy. Front. Nanosci. Nanotechnol..

[31] Aranaz I., Alcántara A., Civera M., Arias C., Elorza B., Heras Caballero A., Acosta N. (2021). Chitosan: An overview of its properties and applications. Polymers.

[32] Rosamah E., Hossain M., Abdul Khalil H., Wan Nadirah W., Dungani R., Nur Amiranajwa A., Suraya N., Fizree H., Mohd Omar A. (2017). Properties enhancement using oil palm shell nanoparticles of fibers reinforced polyester hybrid composites. Adv. Compos. Mater..

[33] Tang C., Zhang Q., Wang K., Fu Q., Zhang C. (2009). Water transport behavior of chitosan porous membranes containing multi-walled carbon nanotubes (MWNTs). J. Membr. Sci..

[34] Manawi Y., Wang K., Kochkodan V., Johnson D., Atieh M., Khraisheh M. (2018). Engineering the surface and mechanical properties of water desalination membranes using ultralong carbon nanotubes. Membranes.

[35] Phao N., Nxumalo E., Mamba B., Mhlanga S. (2013). A nitrogen-doped carbon nanotube enhanced polyethersulfone membrane system for water treatment. Phys. Chem. Earth Parts A/B/C.

[36] Dias P., Quero I., Faraoni J., Palma-Dibb R. (2022). Chemical and morphological characterization of self-etch primers incorporated with nanochitosan. Int. J. Adhes. Adhes..

[37] Ibrahim M., Osman O., Mahmoud A. (2011). Spectroscopic analyses of cellulose and Chitosan: FTIR and modeling approach. J. Comput. Theor. Nanosci..

[38] Bhadra P., Mitra M., Das G., Dey R., Mukherjee S. (2011). Interaction of chitosan capped ZnO nanorods with Escherichia coli. Mater. Sci. Eng. C.

[39] Lawrie G., Keen I., Drew B., Chandler-Temple A., Rintoul L., Fredericks P., Grøndahl L. (2007). Interactions between alginate and chitosan biopolymers characterized using FTIR and XPS. Biomacromolecules.

[40] Thiruppathi M., Senthil Kumar P., Devendran P., Ramalingan C., Swaminathan M., Nagarajan E. (2018). Ce@TiO_2_ nanocomposites: An efficient, stable and affordable photocatalyst for the photodegradation of diclofenac sodium. J. Alloys Compd..

[41] Al-Musawi T., Rajiv P., Mengelizadeh N., Sadat Arghavan F., Balarak D. (2021). Photocatalytic efficiency of CuNiFe_2_O_4_ nanoparticles loaded on multi-walled carbon nanotubes as a novel photocatalyst for Ampicillin degradation. J. Mol. Liq..

[42] Phin H., Ong Y., Sin J. (2020). Effect of carbon nanotubes loading on the photocatalytic activity of zinc oxide/carbon nanotubes photocatalyst synthesized via a modified sol-gel method. J. Environ. Chem. Eng..

[43] Ali H., Motawea E. (2021). Ternary photodegradable nanocomposite (BiOBr/ZnO/WO_3_) for the degradation of phenol pollutants: Optimization and experimental design. ACS Omega.

[44] Zhang J., Yang Y., Sun Z., Zhao D., Gao Y., Shen T., Li Y., Xie Z., Huo Y., Li H. (2023). Ag@BiOBr/PVDF Photocatalytic Membrane for Remarkable BSA Anti-Fouling Performance and Insight of Mechanism. J. Membr. Sci..

[45] Liu J., Wang H., Wu H., Yang Y., Wang C., Wang Q., Jia B., Zheng J. (2024). Research Progress on Zinc Oxide-Based Heterojunction Photocatalysts. J. Mater. Chem. A..

[46] Baig A., Siddique M., Panchal S. (2025). A Review of Visible-Light-Active Zinc Oxide Photocatalysts for Environmental Application. Catalysts.

[47] Jahanban-Esfahlan A., Ostadrahimi A., Jahanban-Esfahlan R., Roufegarinejad L., Tabibiazar M., Amarowicz R. (2019). Recent developments in the detection of bovine serum albumin. Int. J. Biol. Macromol..

[48] Lee E., Moon S., Lee S. (2021). Removal of bovine serum albumin and methylene blue using a hybrid membrane of single walled carbon nanotube-banana peel protein: Fabrication and characterization. Environ. Technol. Innov..

[49] Wang S., Haldane D., Liang R., Smithyman J., Zhang C., Wang B. (2012). Nanoscale infiltration behaviour and through-thickness permeability of carbon nanotube buckypapers. Nanotechnology.

